# Five Social Dynamics Influencing Cholera risks in the City of Goma, Democratic Republic of Congo: A qualitative Study

**DOI:** 10.21203/rs.3.rs-5275711/v1

**Published:** 2024-12-09

**Authors:** Felicien Masanga Maisha, Ndemo Mumbere Mbasa, Kennedy Ulikuwe, Serge Kahatwa, Connie Mulligan, Glenn Morris, Kevin Bardosh

**Affiliations:** Department of Sociology, college of humanity and social sciences, University of Goma; College of Health Sciences, Université Libre des Pays des Grands Lacs; College of Health Sciences, Université Libre des Pays des Grands Lacs; Department of Internal medicine, college of medicine, University of Goma; Department of Anthropology, College of Liberal Arts and Sciences, University of Florida; Emerging Pathogens Institute, University of Florida; School of Public Health, University of Washington

**Keywords:** cholera risks, social, dynamic, public health, Goma, Democratic Republic of Congo, safe water

## Abstract

**Background:**

Cholera remains a major (and increasing) global public health problem. Goma, in the eastern Democratic Republic of Congo (DRC), has been a major cholera hotspot in Africa since 1994 and is currently experiencing one of the largest outbreaks in the world. This article contributes to the existing scholarship on cholera risk by utilizing a variety of qualitative research methods.

**Methods:**

Data were collected between 2021 and 2022 using in-depth interviews, narrative interviews, key informant interviews, transect walks and a participatory mapping workshop, in six areas of Goma. Local understanding of cholera risk stretched across five categories.

**Results:**

First, specific social groups were at increased risk based on age and gender (children, women, elderly), health status (chronic diseases, exposure to cholera treatment centers [CTC]), occupational risks (fishermen, markets) and socio-economic status (impoverished households, refugees, prisoners). Second, cholera risks were framed in relation to broader events such as conflict, population growth, climate change, and volcanic eruptions. Third, the lack of water infrastructure prompted use of unsafe drinking water from Lake Kivu and surrounding lakes. Accessibility of chlorinated water sources was impacted by social connection and cost. Fourth, cholera risk was ascribed to challenges with care seeking and treatment, such as homecare practices, transportation, and substandard practices at CTCs; and issues with implementation of prevention strategies, including vaccination campaigns. Finally, public health outreach practices were viewed as sources of risk by an overemphasis of emergency response teams and insufficient empowerment of communities.

**Conclusion:**

We offer new empirical perspectives on the range of factors that contribute to cholera risk in Goma. These factors should be addressed by implementing diverse strategies, rather than focusing on rapid response interventions. Specifically, development of a safe and reliable water system to treat the chronic nature of cholera infection in the DRC should be prioritized.

## Background

Cholera is a bacterial disease of poverty and socio-political vulnerability. Toxigenic *Vibrio* cholerae O1, the causative microorganism, spreads in areas without safe water, sanitation and hygiene (WASH), breakdowns in public health services, and, often, high levels of overcrowding. Epidemics are associated with humanitarian disasters (including military-political conflict) and natural disasters [1]. In 2022, the most recent year for which World Health Organization (WHO) data are available, there has been a substantial global increase in reported cholera cases and their distribution: 472,697 cases were officially reported. More than double the number of reported cases in 2021 (and probably a gross underestimate of actual global cases[2]; and the number of countries reporting cholera increased from 35 to 44 [1]. Nearly half-a-million cholera cases and 11,897 deaths were reported in the Democratic Republic of Congo (DRC) from 2000 to 2019 [3], although actual numbers could be as high as 280,000 deaths per year [4].

*V. cholerae* O1is transmitted via water or food, or by direct contact with fecal material from patients [5]. Death from cholera may occur within a matter of hours, resulting from severe dehydration due to massive watery diarrhea and vomiting, with mortality rates among untreated patients that can reach 40% [6]. With aggressive intravenous and oral rehydration, administered by trained personnel in appropriately equipped cholera treatment centers (CTC’s), mortality rates can be reduced to < 1% [7]; however, in areas at greatest risk for cholera, facilities, trained personnel, and rehydration supplies may be limited or unavailable. Currently, there is much debate about the best strategies to reduce cholera given the different socio-political and environmental realities as well as the existing public health and medical care systems in the countries most affected [4].

A strategy developed by the *Global Task Force on Cholera Control* (GTFCC) aims to reduce global cholera deaths by 90% and eliminate the disease in 20 countries by 2030 [4]. This approach attempts to reconcile divergent ideas about the prioritization of long-term WASH infrastructure investments with short-term rapid emergency response that have traditionally generated very different ideas of how to control cholera. Based on this work, Ratnayake et al. [8]have described the development of a rapid response strategy in Goma, eastern DRC, a major cholera “hotspot” in Africa, which included a ring strategy integrating oral cholera vaccination (OCV), antibiotic prophylactic, and household WASH supplies.

Goma is situated on the northern shore of Lake Kivu and shares a border with Rwanda. Goma has long been a site for major global humanitarian assistance due to war, poverty, natural disasters, epidemics, and poor governance. In 1994, Goma became the epicenter for one of the most devastating modern cholera epidemics, with an estimated 50,000 deaths [9]. Goma came under attack by the Rwanda-backed M23 rebel group in 2008 and was temporarily seized by this group in 2012, before being driven out by Congolese and UN troops. In 2021, the M23 group sprang back to life, and currently controls substantial areas of North Kivu province. Goma is situated next to Nyiragongo Volcano, where eruptions occurred in 2002 and 2021[10];[11], with the 2021 eruption resulting in displacement of an estimated 400,000 persons. The second largest Ebola epidemic occurred between 2018–2020 in eastern DRC, including in Goma [12]. Taken together, it is currently estimated that there are close to 1 million displaced persons in camps surrounding Goma, with major cholera outbreaks occurring within these camps. [10]; [11].

In DRC, poverty and food insecurity are widespread, with an estimated 60% living below the poverty line in North Kivu. Furthermore, approximately 70% of Goma in habitants do not have easy access to clean water [13].

In this paper we present the results of a multi-pronged qualitative research study focused specifically on Goma on the shore of Kivu Lake which would play a certain role in the transmission and the close epidemic cholera outbreak in this city [14]. Goma is also a setting in which there was ongoing implementation of various intervention strategies respond to the endemicity of cholera. We used the qualitative approach to explore for an extensive understanding of the probable social dynamics driving the cholera in the city of Goma. This ethnographic approach helped our team to collect empirical perspectives from the cholera effected communities to inform public health policy in Goma, with application to other places in the region in which cholera continues to be a major public health concern.

## Methods

The study was conducted in Goma between June 2021 and September 2022 and broadly explored the relationships among demography, socio-political and environmental events, urban infrastructure, and public health response related to cholera. Most study participants referred to cholera risks as “vulnerabilities” to reflect the broad range of relevant factors that impact cholera infection, transmission, and treatment – in this paper, we use the term “risk” since it is more commonly used and recognized in peer-reviewed publications.

### Study context

Goma is situated on the northern shore of Lake Kivu and shares a border with Rwanda. Goma has long been a site for major global humanitarian assistance due to war, poverty, natural disasters, epidemics, and poor governance. In 1994, Goma became the epicenter for one of the most devastating modern cholera epidemics, with an estimated 50,000 deaths [7]. Goma came under attack by the Rwanda-backed M23 rebel group in 2008 and was temporarily seized by this group in 2012, before being driven out by Congolese and UN troops. In 2021, the M23 group sprang back to life, and currently controls substantial areas of North Kivu province. Goma is situated next to Nyiragongo Volcano, where eruptions occurred in 2002 and 2021[8];[9], with the 2021 eruption resulting in displacement of an estimated 400,000 persons. The second largest Ebola epidemic occurred between 2018–2020 in eastern DRC, including in Goma [10]. Taken together, it is currently estimated that there are close to 1 million displaced persons in camps surrounding Goma, with major cholera outbreaks occurring within these camps. [8]; [9].

In DRC, poverty and food insecurity are widespread, with an estimated 60% living below the poverty line in North Kivu. Furthermore, approximately 70% of Goma in habitants do not have easy access to clean water [11].

### Site selection

We selected six areas of Goma with a total estimated population of 168,654 people: four administratively defined health areas (Nzulo, Buhimba, Kiziba and Kasika) as well as the Katindo military camp and the Munzenze prison[1]. These latter sites were selected because they are peripheral areas known to be cholera “mini-hotspots.” Three areas (Buhimba, Kiziba, and Katindo) have cholera treatment centers (CTCs) in their administrative boundary while the others (Nuzulo, Kasika, and Munzenze prison) have small medical units that regularly report and treat cholera[2].

**Figure 1:**
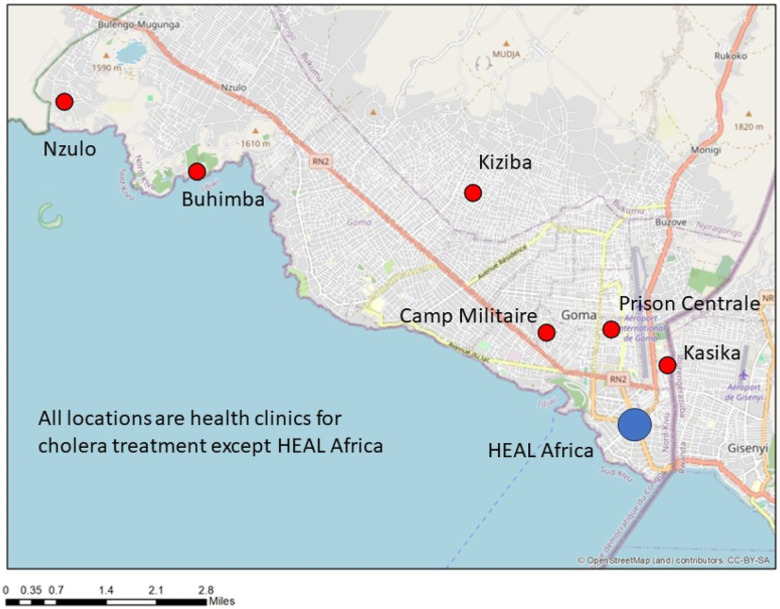
Map of Goma showing the data collection sites

Table 1 is about the participants of this study by gender and by method; Figure 1 shows the location of the major centers of cholera treatment in Goma, which coincide with our study locations and HEAL Africa, a major medical facility supported by non-governmental organizations (NGOs), which served as our operational base and the site for the participatory mapping. In Table 2, we provide Cholera cases from 2016 to 2021 in the city of Goma.

### Research methods and analysis

We conducted 21 in-depth interviews, 12 interviews with key informants, 7 narrative interviews, 12 focus group discussions (FGD)[3], 5 transect walks, and one participatory mapping workshop[4]. We used purposive sampling to select our study participants based on their availability during data collection and their role or occupation (local leaders, government medical staff, and members of community-based organizations and NGOs). Three different interview guides were developed for the in-depth interviews to explore a range of topics including community understanding, behaviors, and practices related to cholera transmission, treatment, and prevention as well as community experiences and responses to cholera control and prevention. Key informant interviews were conducted with community leaders and local health experts to explore the range of cholera activities in Goma including governance, delivery, and policy issues, as well as the strengths and weaknesses of cholera interventions. Narrative interviews generated local narratives of environmental and social risk factors and changes related to cholera. In each of the six study locations, at least two FGDs and one transect walk excluding in the Munzenze Prison were held. The first FGD focused on community understanding, behaviors, and practices related to cholera transmission, treatment, and prevention measures. The second FGD focused on community experiences and responses to cholera control and prevention interventions. Participatory mapping was conducted in the meeting room of HEALAfrica with actors from the health sector, public institutions involved in water supply and waste management, and local and international NGOs[5]. Activities included event timelines, service delivery analysis, and risk factor mapping.

Data were collected in Swahili and French, which are the most common languages spoken in the study area[6]. Notes and audio-recordings were used during fieldwork, which were transcribed into French and entered into Microsoft Excel. Data analysis was based on thematic analysis using a codebook. A draft codebook was used and was refined over three cycles of team data analysis.

[1] Munzenze is the main prison in Goma and we included this location to assess risk within this special patient population. For ethical reasons, we did not include prisoners among our direct research participants. We chose to conduct interviews with guards, managers and the health staff of the prison to better understand cholera risk within this specific population, given that the Munzenze CTC was regularly reporting cholera cases.

[2]It is generally known that a CTC is bigger in terms number of beds and the complexity of services offered but in Goma, sometimes it is difficult to differentiate a CTC from a UTC because during large cholera epidemic outbreaks, capacity of all UTCs is enhanced with additional beds and services to accommodate caseloads. As case numbers decline, the additional beds, staff and services are taken away and the big CTC becomes a small cholera treatment unit.

[3] We conducted 2–3 FGDs per site except in the prison where we did not conduct any interview with prisoners. 8–10 people participated in each focus group that took place in a location provided by a local leader.

[4] Primarily two members of our research team who are fluent in the Congolese dialect of Swahili and who have extensive experience with fieldwork in eastern DRC conducted interviews.

[5] Participants were selected from the 6 sites and included community leaders in different sectors (health, education, religion, business and local political leaders.

[6] Swahili is the major language spoken in eastern DRC. French is the official language of the country and key informants preferred to be interviewed in French.

## Results

**Figure 2:**
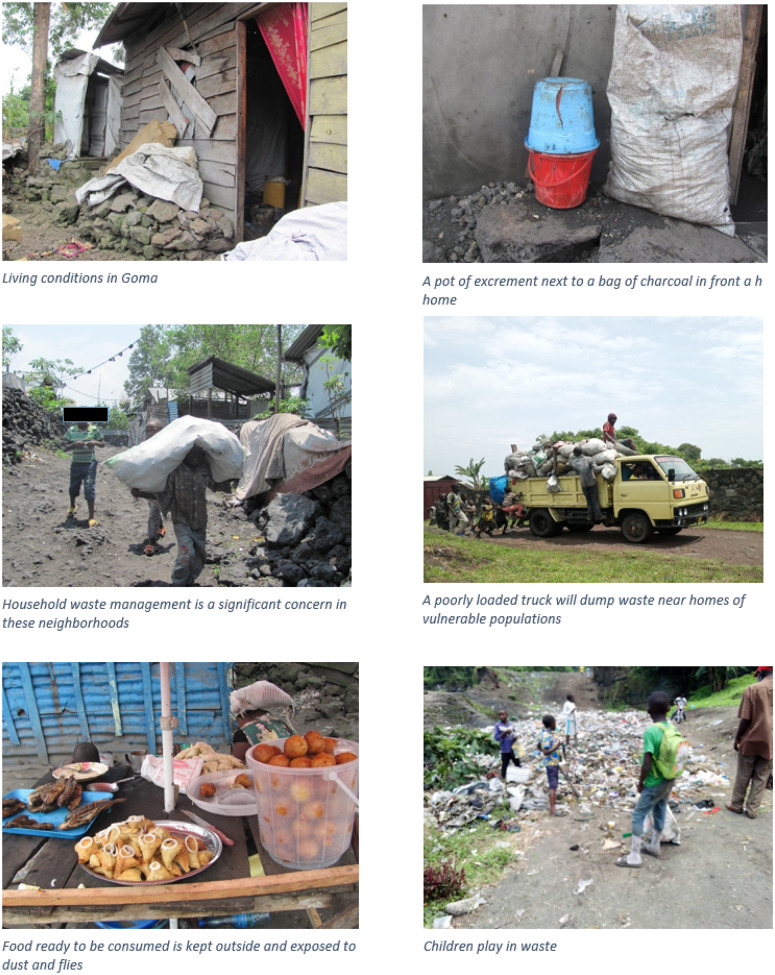
Fieldwork images of cholera risk in Goma, DRC

Our results are divided into five sections. These explore 1) high risk social groups, 2) conflict and environmental events, 3) water infrastructure, 4) cholera treatment and 5) community-based outreach and prevention. Figure 2 provides visual context for these findings from our fieldwork. Implications for cholera control and prevention are outlined in the discussion section.

### High risk social groups

Cholera risk was associated with specific social groups that was related to interactions between individual vulnerabilities, behaviour and the environment. High risk social groups were divided by age and gender, health status, occupation, and socio-economic status.

#### Vulnerable age and gender groups

Children, women and the elderly were widely considered to be at greater risk. For younger children, risk was related to play and eating behaviors (Figure 2) and for older children risk; it was related to hand-washing and food hygiene. Infants were considered higher risk when growing teeth, crawling, and learning to walk. Infants were believed to be affected by the diet and hygiene of a lactating mother while women were at increased risk because of their care roles in the household, including caring for sick family members and washing clothes. The younger children were believed to be more susceptible for two reasons: they are sometimes unable to care for their own hygiene and they face challenges with water accessibility since they must find someone to fetch water for them. Elderly people also regularly keep water for much longer than other groups, which was believed to increase chances of contamination.

#### Health status and health staff exposure

Chronic diseases, including HIV and diabetes, as well as malnutrition were believed to increase risk for serious cholera infection outcomes. CTCs were considered to be potential ‘hotspots’ for cholera spread through exposure of caregivers, staff and visitors, especially hygienists who handle soiled clothes and clean CTCs.

#### Occupation

Community members identified two occupational factors believed to increase cholera risk: markets and fishing. Public markets were believed to be sources of cholera for three reasons. First, the lack of toilets (both formal and informal) created unhygienic conditions, including public trashcans and bags with feces. Second, people who go to markets often consume ‘quick food’ (including benye, sambusa and grilled fish) which is not always safe due to the high number of flies (Figure 2). Third, when there is heavy rain, market sellers sometimes leave their goods on the ground (and cover them with plastic bags) where the runoff water is believed to contaminate the food.

Cholera risk among fishermen was linked to their constant presence around lake water and their propensity to drink untreated water from Lake Kivu: *“we do this when we are far from the side of the lake, far from the coast, in the big lake”* (In-depth interview, fisherman, 2021). In addition, fishermen were widely thought to be more likely to lack latrines and consume unclean foods from the market.

#### Socio-economic status

Cholera was overwhelmingly associated with poverty and poor households. Household waste management is a serious problem (Figure 2). When houses are built on volcano lava, as in the case of Nzulo health area, it is difficult to dig simple pit latrines. In some areas, latrines are provided by NGOs but these benefits only accrue when the NGO is operating in the area. In the Katindo camp, houses are built on lava and Quarter 4 is known as a cholera hotspot due to lack of latrines and unhygienic conditions, such as using pots for sanitation, which allows flies to proliferate.

“People who are more at risk of catching cholera are in the Katindo camps, especially people living in Quarter 4. Ten households for a toilet built from rubbish. It is at the same time the place where we throw the garbage, and at the same time we empty the pots [there]. There is a bad smell, lots of flies, the houses are too close together.”

(FGD, community relays, Katindo camp, July 2021)

Poverty was also believed to create a ‘way of life’ where a household would not be able or willing to maintain standards of hygiene, food safety, and cleanliness. People who rent land or homes were also believed to be at increased risk because they have no toilets and defecate in the bushes.

“Those who defecate in the open, you can find a person whose toilet is already full, he has no means, he has not yet had the means to empty it or build another one…he goes to arrange himself one evening, in a corner of someone else’s house, and leaves the dirt there”

(FGD, local leaders, Kasika, June 2021)

Conditions in the Munzenze prison were viewed as an important source of cholera risk in Goma. There is limited access to water and the septic tanks are only emptied every 2 to 3 days. The number of prisoners exceeds the capacity of the prison by a significant amount. This overcrowding was blamed for the spread of cholera, as well as poor food hygiene and lack of sanitation. Prisoners also suffer from high rates of HIV, tuberculosis and malnutrition.

“I’ve been here for 4 years, cholera is too visible here, and I think the cause is the large number of people in the prison. If we don’t work hard to keep clean in the prison, we would bury every day because cholera kills…people who are in the cells live in very poor conditions and when they are transferred here, they often come with bad smelling clothes, they spend several days without even washing. So when they mix with the others here, they contaminate themselves, and maybe when they arrived there was no water, they sleep in the dirt.”

(FGD, health staff, Munzenze prison, 2021)

### Conflict and environmental events

A second way that cholera risk was understood in Goma related to socio-political and environmental factors. This included conflict, population growth, climate change and volcanic eruptions.

#### Ongoing conflict

Ongoing armed conflicts in and around Goma have presented a major challenge for public health. Conflicts provoke a rural exodus and migration to the city and also disrupt roads that influence the development of the region including the ability to seek medical care and supply clean water. It also means that internal refugees are a common occurrence, which puts pressure on land and urban infrastructure. Yet some informants stressed the positive trends in development and urbanization in Goma including a “change in mentality” that is reducing cholera risk.

“People start to change when they are in contact with the city…In the past, in terms of education, people said that sending children to school is a waste of time, but today if you have children at home, others call you and ask you to go to school….today people are starting to be clean. Several revival churches have emerged. On the economic level, people [now]…focus on commercial activities…the tribalist behavior is changing in order to live together.”

(In-depth interview, local leader, Kiziba, June 2021)

This optimistic perspective was counteracted by more pessimistic viewpoints that stressed increasing food insecurity, insufficient arable land, and population growth as risk factors for cholera. Population growth in some places, such as Nzulo, is increasing while infrastructure such as drinking water and hospitals are not available. Population growth is putting severe pressure on farming, reducing plot land per household. New buildings often do not have latrines due to a lack of resources and lack of property ownership laws.

“The people of Lushagala are getting ill due to the lack of hygiene, especially since there is no pure water…the toilets are non-existent because everyone is keeping a plot and the owners of construction sites refuse to have toilet holes dug in their plots and they say to themselves “how am I going to dig a hole in someone else’s plot and then be kicked out?”

(FGD, political-administrative authorities, Buhimba, 2021)

With increased migration to the cities, many young people do not have jobs. Unemployment was believed to be related to increasing crime and insecurity on the periphery of the city, especially in Nzulo, which reduces people’s willingness to invest in their homes including WASH infrastructure.

“There is a problem of insecurity. Thieves during the night visit people and loot at the point that it impoverishes people even more, and others are afraid to move to these areas because of this insecurity or after having bought a plot, when he hears that there is insecurity, he decides to stay in [urban] Goma as long as he has all the possibility of building. This leads to the spaces remaining uninhabited and used as a place of waste disposal.”

(FGD, local leaders, Buhimba, June 2021)

#### Environmental factors

Water from Lake Kivu was believed to be contaminated with germs, including cholera, by laundry, bathing, defecation, corpses, and the presence of waves on Kivu Lake. Water movements bring a lot of dirt back to the edge of the lake, which contribute to water pollution. Contamination was also believed to include the washing of clothes from children and others who are suffering from diarrheal diseases.

“We are in April, so soon there will be waves at the level of the lake and the microbes will be transported along the lake and consumed by the population which does not accept to chlorinate the water. Waves increase a lot each April of the year.”

(Narrative Interview, teacher, Nzulo, 2021)

Natural disasters are viewed as a cause of cholera epidemics, including the volcanic eruption of May 2021. This was ascribed to overcrowding, hygiene conditions, and population movements. Some patients had been forced to leave the CTC when they saw people fleeing the city from the volcanic eruption while they were still sick from cholera. Cholera risk was also associated with the presence of dead bodies on Lake Kivu, which was believed to contaminate the water. Cholera outbreaks were considered to be more frequent during the dry season since heat creates a shortage of drinking water and requires more people to seek water directly from Lake Kivu. Climate change is disturbing local farming.

“The consequences of this climate change are serious. There is famine. In August the parents can no longer find beans to give to their children. By June, we are going under a blazing sun when all the crops are already dried before the flowers even appear on them.”

(Narrative interview, former teacher, Nzulo, April 2021)

### Water infrastructure

A third cause of cholera risk was associated with water infrastructure and lack of safe and clean water. Lake Kivu is the only source of water in Goma. However, REGIDESO[7] distributes treated water from Lake Kivu but only a very limited population have access to it. Thus, for the majority of people two options are available: drink chlorinated or unchlorinated water from Lake Kivu.

#### Unchlorinated water

Beliefs and attitudes regarding unchlorinated water and cholera risk generally fell into two categories: those who believed unchlorinated water was a major risk factor for cholera and diarrheal diseases because it came from the lake and those who did not. This belief was related to many different perceptions about immunity and health, generally associated with the way individuals obtained their water.

“People have no choice, they take knowing that there is dirt but we take it like that. And that’s okay! But also, there are people who don’t know that there is chlorine that he should put in the water….they think it’s normal to take [lake water]. Others say black people don’t die of germs.”

(FGD, community outreach team, June 2021)

“There are those who even refuse aquatabs [chlorine tablets] because they say that when my body is already used to aquatabs and I run out of them, I will risk getting sick because my body will no longer support microbes so it does not accustom the body to aquatabs because they are transient.”

(Interview, medical staff, Nzulo, 2021)

Daily life requires one to ignore the association between cholera and lake water due to poverty. For example, the Buhimba health area is supplied with drinking water from the company Yme Jibu and there are standpipes or tap stations placed along the streets to help people draw water for payment by canister, but few people have money to pay these fees. Boiling water is unrealistic due to the cost of embers and firewood.

“Here in Nzulo we consume dirty water. We take water drawn directly from the lake and sometimes some partners have come to help us with chlorine and especially this partner comes in case of epidemic only. But really the water we drink here is very dirty.”

(FGD, community relays, Nzulo, 2021)

During the transect walk in Buhimba, participants stated that people who live near the green lake (Lac vert) have noticed problems with water chlorination that they ascribe to green algae and salinity. On the other hand, some households also collect rainwater if water from tanks is not available, especially during the rainy season.

#### Chlorinated and safe water

According to data from our participatory mapping and transect walks, people have access to chlorinated water in Goma by: 1) Bitanké (community tanks built with cement) 2) dealers known as REGIDESO (a public company), 3) water supplied in tanks distributed by NGO trucks and 4) city pipes.

Furthermore, water supplies through the Bitanké involve a social hierarchy that mediates access to water. Intimidation and influence peddling impact access to water at these sites, which are managed by the Congolese police. Sometimes, too, the police can help their friends obtain water without being worried, as they are the ones who manage the standpipes.

“People wake up at 4 a.m. to keep the line but there are people who arrive there, they are called “capable”, another person who arrived at 6 o’clock will draw in front of you and people will start fighting. People are fighting there as they tell us it is state water so there are police collaborating with the capable and people of the community cannot speak if it is the capable who draw.”

(FGD, water dealers, Kiziba, June 2021)

Water supplies are also provided by tanks. Trucks with tanks bring water to different corners of the city. This water is kept in tanks made locally by certain dealers who supply water to the population. Tanks can be disrupted by conflict and natural disasters. For example, the pipeline that Mercy Corps relies on to fill its tanks in UNIGO[8], which supply Bushagara to Munigi, was destroyed by the volcanic eruption.

“Here it’s the tanks first, because there is a water cut on the taps. It can happen that at 11 a.m. there is no more water while many people have not yet been served. This is the reason why everyone relies on tanks. The tanks are many here, we just see the car that comes to pour water and we do not know if they wash the tanks and if they put the medicine [chlorine]. ”

(Indepth interview, community relay, 2021)

Water is also provided by the company REGIDESO, which has pumping stations installed along Lake Kivu to supply chlorinated water to households through a pipe setup.

“Resellers can also draw water from the lake in sites such as REGIDESO (where a can is bought at 200 FC), the beach, Kituku, Caracciolini, and CCLK. The person in charge gives you a slip which certifies that the water contains chlorine. We have only one terminal in Kiziba 2. Households also arrive at these sites with their own cans.”

(FGD, water dealers, Kiziba, June 2021)

Finally, roughly 30% of people in the city center of Goma are in households that rely on piped water supplies. Establishing a piped water system and maintaining it is a viewed as a political act.[9] In his 2006 election campaign, former president Kabila included the access of water among his five priorities. The promise of clean water as a key government program to supply water and electricity to the majority of the Congolese people did not become a reality.

### Cholera treatment

A fourth layer of cholera risk in the Goma area was related to challenges with seeking care and treatment. This factor included homecare practices, transportation, and substandard care practices at CTCs, such as a lack of medical supplies, water and sanitation.

Although amoeba, typhoid fever, and other diarrheal diseases are widespread, most people prefer using indigenous treatments first and they will think about modern treatment or going to a CTC only later.

“There are times when you feel diarrhea when you have abdominal pain and you go to the toilet… You can cut the leaves as long as it’s something else; when the light stools start coming down and you go to the toilet and so the most familiar sign is how diarrhea attacks someone when the stools go down there you realize it’s diarrhea and you go running fast look for herbs to eat and if these herbs do not relieve you and if the diarrhea continues then you have to go to the hospital quickly.”

(FGD, local leaders, Buhimba, June 2021)

Participants mentioned an important culture of homecare in Goma and the difficulties with isolating a family member even if cholera is suspected. Access to community-based oral rehydration therapy (ORT) to use at home was not available. Diarrheal illness is typically treated at home with embers or ashes, the roots of papaya, aloe, garlic, prayer, coffee, and herbal medicines.

“Another method is using coffee. We boil it without sugar; we give it to the patient, just to calm the diarrhoea. Others use wheat flour, put a little in a glass, mix it with a little water, then the patient drinks it, just to calm the diarrhea before arriving at the hospital.”

(FGD, communtuy relays, Katindo, July 2021)

Sometimes home treatment includes concerns about a child being bewitched and involves prayer and spiritual practices. Once these efforts have been used with no success, a family will balance the need to seek medical care with the cost, family income, and concerns about quality of care. During the height of the COVID-19 pandemic, some people reported being reluctant to seek care at CTCs due to concerns about being transferred to a COVID-19 center.

In some areas far from CTCs, local motor-bikers are believed to discriminate against transporting patients who are suspected of having cholera, for fear of being contaminated. Other difficulties include traveling at night due to the security situation.

“I found that the child was already in a critical situation, I had to look for the motorcyclist at night, I asked them to carry the child and that I will do whatever they can ask; the first person told me that his motorcycle has no lights even though it was not true.”

(Narrative interview, former teacher, Nzulo, 2021)

Even if a patient does go to a CTC, they may encounter a lack of medical supplies when there is no assistance from NGOs, which was noted to be especially the case at the Nzulo CTC. Drugs that are frequently missing from CTCs include intravenous infusions for rehydration. During a cholera outbreak, NGOs will support CTCs by paying staff salaries and providing food to patients and their caregivers. However, when the outbreak ends, NGO support can be erratic and staff are paid irregularly. Most CTCs do not have electricity, which makes caring for patients at night problematic. There is often insufficient personal protective equipment during periods of cholera epidemics for nurses and community relays. In the Kiziba CTC, some noted the lack of water and sanitation.

“There is no water in the CTC and there are people spraying their defecation on the wall…that makes it dirty…patients share water. This can create problems and can be a source of contamination and moreover some accompanying patients are afraid to go and draw water from the CTC and prefer to go fetch water outside because there are bad smells.”

(FGD, medical staff, Kiziba, 2021)

### Community-based cholera prevention outreach

A final area of cholera risk related to the practices and activities of public health stakeholders in planning and outreach. Our study participants questioned the sustainability of NGO activities, emphasized the lack of coordination, and believed that community outreach teams lacked capacity. One way these sentiments were expressed was in widespread scepticism about the coverage and acceptance of cholera vaccination.

Many community members and leaders emphasized their frustration with the effectiveness of NGO activities on cholera in Goma. There are many different NGOs that operate in our study sites and work in cholera prevention: MSF (Doctors Without Borders), Red Cross, Yme Grands Lacs, and Mercy Corps. Given socio-political realities in Goma, NGO interventions are viewed as partial, temporary, and focused largely on emergency situations. These factors make it difficult for NGOs to contribute to the sustainable development of the city.

The short-term effectiveness of NGOs was noted above in terms of medical supply shortages and unreliable access to water at the CTCs. Other examples included the lack of hygiene promotion, materials at schools, weak evaluation and follow-up, and a neglect of longer-term strategies. For example, Mercy Corps intervened in recent years in Nzulo by building toilets for local residents but we found that few beneficiaries are able to empty the toilets when they are full, again reflecting the temporary effectiveness of NGOs.

“MSF’s withdrawal sometimes disrupts cholera treatment activities… at any time when it withdraws, it always leaves the center to manage itself and the government is not able to take over”

(In-depth interview, medical staff, Nzulo, September 2021).

Participants emphasized the important role of community relays, who can refer cases from the community, although some are unable to maintain their own personal WASH standards. Community relays play a major role in sensitizing local communities to use health facilities if any illness occurs. They work permanently in the area but are only supported by NGOs during short project periods which poses a problem for the development of a sustainable community-based system. The volunteer work of community relay sometimes discourages them because they are not paid.

Concerns about NGO activities and sustainability in cholera prevention were also reflected in widespread scepticism about cholera vaccination coverage and acceptance. In 2019, a large-scale oral cholera vaccine (OCV) campaign was funded by WHO/GAVI from the global OCV stockpile. This campaign occurred across North Kivu during both an active Ebola and measles epidemic [12]. Community members raised several issues about this campaign in the Kiziba, Nzulo and Buhimba health areas, including lack of advertising and training, inadequate follow-up for the second dosage, rumours about vaccine side effects (e.g. infertility and shortened life expectancy), and concerns that children accidentally received multiple doses because of a lack of proper documentation. It is important to note that we are not able to confirm the accuracy or extent of these issues, but they do reflect real concerns among Goma inhabitants that possibly limit the future effectiveness of NGO activities.

The critique about NGOs also involved criticisms about governance challenges and gaps between public health partners and the government.

“The responsibility of the health center should include the power to decide how organizations intervene according to the real needs of the community. They have limited decision-making power in the fight against cholera. The decision-making power is not found at the local level where the actors intervene”

(In-depth interview, medical staff, Nzulo, 2021)

Specific recommendations were made to leverage political advocacy and community action to improve water supplies. For example, advocacy with the company *Yme Jibu* to increase the number of tap stations supported by local actors in Buhimba in order to facilitate geographical accessibility to water. Informants also stressed the importance of community action including local leaders and the *Salongo* system, which involves weekly household cleaning activities but could also organize the community sanitation work if necessary.

7 REGIDESO is the public company of water distribution in the DRC. It used to be the only company providing water to the DRC but recently, because of the obvious weaknesses of REGIDESO, the DRC government allowed private companies to get involved in water distribution. Today, Yme Jibu is involved in the business of water distribution. However, both Yme jibu and REGIDESO are not able to fulfill the water needs of the population of the city. In addition, there is a general perception in the population that the water from the lake, even treated by REGIDESO, is not clean because of the savor of salt of this water.

[8] Name of a neighbourhood in Kiziba Mercy corps, an American NGO, is involved in building water infrastructure in this part of Goma.

[9] The public company of water distribution in the DRC. It used to be the unique company providing water to Congolese but recently, because of the obvious weaknesses of REGIDESO, the government of DRC allowed private companies to get involved in water distribution

## Discussion

Our qualitative study identified specific social groups, divided by age and gender, health status, occupational and general socio-economic status, believed to be at higher cholera risk in Goma. Like other social studies in Africa [13], specific water and food contamination pathways were associated with particular socio-behavioural vulnerabilities. While case-control studies [14]; [15];[16];[17] may help identify sources of a cholera outbreak, our approach has its advantages. We were able to identify specific risk factors at public markets, fishing communities, in a prison, poorer neighbourhoods without latrines or safe water, and cholera treatment centres.

In our study, cholera risk was also understood in relation to broader socio-political and ecological factors. Other studies have explored some of these risk factors in DRC. Kayembe et al. [18] found an association between conflict events and the geographic spread of cholera across DRC. Bompangue et al. [19] found clustering of cases in lakeside towns but did not find that epidemics systematically followed complex emergencies. Finger et al. [20] found that climate anomalies and rainfall were correlated to cholera cases. Batumbo Boloweti et al. [21] found relationships among clinical cholera cases in the community; environmental *V. cholerae* in Lake Kivu and locally harvested fish (albeit without testing for serotype or toxigenicity of strains); and the intensity of volcanic activity, with potential linkages to water temperature and other physiochemical properties of lake water. We found that local perceptions of cholera risk are strongly related to Lake Kivu activities including doing laundry, bathing, drinking water, defecation, corpses, volcanic activity, dry season behaviours, lake waves, and microbes that are believed to be endemic to the lake. Further microbiological data is needed to explore whether an environmental reservoir of toxigenic *V. cholerae* O1 exists in Lake Kivu and its possible role in outbreaks.

We found major shortcomings in water access in Goma and widespread use of unchlorinated water from Lake Kivu. These findings echo research by Ciraane et al. [22]. An irregular and unreliable patchwork of piped sources, Bitanké, dealers, rainwater, and NGO tanks supply water to a growing population, predicted to double by 2030 [23]. Women and children spend considerable time waiting in lines and paying high prices for water.

Jeandron et al. [24] argued that a reliable water system, especially a tap network, is likely to be the best long-term strategy for reducing cholera cases in DRC. Behavioural programs must account for the realities of water and sanitation insecurity in Goma, including land use issues. Some of our participants believed that using chlorine intermittently, without sustained supplies, could increase individual susceptibility to diarrheal diseases since ‘natural’ water builds-up immunity to pathogens. The algae and salinity of green lake water was believed to prevent ‘proper’ chlorination. Both of these local perceptions have relevance for rapid cholera response strategies that distribute chlorine.

Our research identified homecare practices, discrimination of motorcycle taxis, and substandard care practices at CTCs as barriers to cholera treatment. Many cholera deaths occur in the community or before arrival at a treatment center [25]. Not everyone with severe diarrheal has cholera, although lay differentiation is difficult. ORT was unavailable at the community-level and increasing the availability of ORT may help patient clinical outcomes. We found widespread concerns about the lack of medical supplies at CTCs and substandard WASH infrastructure, which should be addressed. A recent study in Kinshasa found low levels of cholera preparedness among frontline healthcare centers [26].

Cholera has long generated popular blame directed at government and humanitarian inadequacies. Cholera outbreaks are not only a public health crisis but also create parallel political, economic and social crises, inserting itself into debates about citizenship, rights, and expectations about public services [27]. We found that cholera control intersects with community perceptions and feelings about inadequate NGO programs and public services. As a center for humanitarian action, Goma has long challenged humanitarian models of governance and disaster recovery [9]. We found challenges with the over-emphasis on emergency response, duplication of activities, lack of capacity for community health agents, and scepticism about vaccination campaigns. Improving capacity for planning, the involvement of community relays, monitoring, evaluation and intersectoral action are needed to better address cholera in Goma.

A new rapid response approach, known as case-area targeted intervention (CATIs)[10], has been proposed in DRC including Goma [6]; a ring approach that integrates WASH, OCV, and antibiotic chemoprophylaxis but excludes water improvement. The CATI approach has been implemented in Haiti and in Africa since 2017 [28], but clear documentation of its efficacy as an isolated intervention in the absence of a more comprehensive public health approach is lacking. Other rapid approaches have been developed, such as by [29] in DRC to reduce intra-household transmission in DRC. The emergency CATI attempts to uproot conventional wisdom about the feasibility of cholera control and elimination in fragile states. For examples, there are concerns that emergency strategies narrowly directed against one pathogen may detract or stymie local water infrastructure and other system-strengthening goals. As CATI is scaled-up in the DRC, further work should evaluate the approach and explore the temporal and spatial dynamics of cholera in relation to the social vulnerabilities identified in this qualitative assessment.

[10] UNICEF, based on preliminary results from Haiti, introduced the CATI approach in the DRC in early 2020. The DRC RedCross has played a key role in implementing the approach. The improvement of water infrastructure is not part of the CATI because the approach has to be quick, used in the immediate time after a cholera infection reached a household. As part of the CATI approach, households in Goma were given chlorine tablets.

## Conclusion

Cholera has become an almost daily reality in the lives of Goma residents. This qualitative study identified the social dynamics influencing cholera in this city expanding the understanding of the almost permanent nature of the cholera epidemic in Goma. Policymakers and other community actors including humanitarians should consider this by implementing diverse strategies, mostly involving the affected communities rather than focusing on rapid public health outreach response interventions. Lastly, the long-term development and the maintenance of a safe and reliable water infrastructure in the city will contribute to the reduction of the chronic nature of cholera infection in the city of Goma.

## Figures and Tables

**Table 1 :** Research participant characteristics

	Methods	Focus Group Discussion	In-deph Interviews	Key Infor. interviews	Participatory mapping	Narrative interviews	Transect Walk	Total
	N	85	21	12	20	7	20	165
Age	Median	38,0000	39,0000	44,5000	36,0000	43,0000	37,5000	38,0000
	Min	18,00	26,00	29,00	28,00	30,00	24,00	18,00
	Max	75,00	72,00	56,00	58,00	69,00	59,00	75,00
	% Total	51,5%	12,7%	7,3%	12,1%	4,2%	12,1%	100,0%
Gender	Female	45 (27,3%)	6 (3,6%)	4 (2,4%)	7 (4,2%)	1 (0,6%)	11 (6,7%)	74 (44,8%)
	Male	40 (24,2%)	15 (9,1%)	8 (4,8%)	13 (7,9%)	6 (3,6%)	9 (5,5%)	91 (55,2%)
education	No formal education	5 (3,0%)	0 (0,0%)	0 (0,0%)	0 (0,0%)	0 (0,0%)	0 (0,0%)	5 (3,0%)
	Primary school	10 (6,1%)	1 (0,6%)	1 (0,6%)	8 (4,8%)	0 (0,0%)	0 (0,0%)	20 (12,1%)
	Secondary school	56 (33,9%)	9 (5,5%)	4 (2,4%)	1 (0,6%)	4 (2,4%)	15 (9,1%)	89 (53,9%)
	University	14 (8,5%)	11 (6,7%)	7 (4,2%)	11 (6,7%)	3 (1,8%)	5 (3,0%)	50 (30,3%)

Source: present research data

**Table 2:** Cholera cases in Goma (2016–2021)

Reported cholera cases in Goma	2016	2017	2018	2019	2020	2021
	712	6859	624	4019	1846	824
Reported deaths	1	4	0	3	2	2

Source: Unpublished data, North Kivu health division, branch of the DRC Ministry of Health

**Table 3:** Operational cholera treatment centers at study sites (2022)

N°	CTC	Supporting partner	Staff of the CTC	Number of beds
1	Kasika	MSF	4 (2 nurses & hygienists)	18
2	Katindo	WHO	6 (4 nurses & 2 hygienists)	20
3	Prison Munzenze	UNICEF/WHO	5 (1 doctor, 2 nurses & 2 hygienists).	20
4	Kiziba	UNICEF/WHO	4 (2 nurses & 2 hygienists)	18
5	Buhimba	MSF	4 (2 nurses & 2 hygienists)	20
6	Nzulo	MSF	4 (2 nurses & 2 hygenists)	8

Source: DRC Ministry of Health

## Abbreviations

CATI: Case Area Targeted Intervention
CTC: Cholera Treatment Center
DRC: Democratic Republic of Congo
FGD: Focus Group Discussion
GTFCC: Global Task Force on Cholera Control
MSF: Medecins Sans Frontieres (Doctors without borders)
ORT: Oral Rehydration Therapy
OVC: Oral Cholera Vaccination
WASH: Water, Sanitation and Hygiene
WHO: World Health Organization
